# Emergent Techniques for Transporter and Receptor-Based Imaging and Interventional Molecular Imaging

**DOI:** 10.1155/2018/7615308

**Published:** 2018-12-20

**Authors:** Paulo Henrique Rosado-De-Castro, David Yang, Hwan-Jeong Jeong, Kazuma Ogawa, Marcos Fabio Henriques DosSantos, Skye Hsin-Hsien Yeh

**Affiliations:** ^1^Institute of Biomedical Sciences, Federal University of Rio de Janeiro, 21941-590 Rio de Janeiro, RJ, Brazil; ^2^Department of Radiology, Federal University of Rio de Janeiro, 21941-913 Rio de Janeiro, RJ, Brazil; ^3^D'Or Institute for Research and Education, 22281-100 Rio de Janeiro, RJ, Brazil; ^4^Vyripharm Biopharmaceuticals, 77021 Houston, Texas, USA; ^5^Department of Nuclear Medicine, Molecular Imaging and Therapeutic Medicine Research Center, Chonbuk National University Medical School and Hospital, Jeonju, Jeollabuk-do 54896, Republic of Korea; ^6^Research Institute of Clinical Medicine, Biomedical Research Institute, Chonbuk National University Hospital, Chonbuk National University, Jeonju, Jeollabuk-do 54896, Republic of Korea; ^7^Institute for Frontier Science Initiative, Kanazawa University, Kakuma-machi, Kanazawa, Ishikawa 920-1192, Japan; ^8^Brain Research Center, National Yang-Ming University, 11221 Taipei, Taiwan

The growing field of molecular imaging has allowed noninvasive *in vivo* tracking of pharmacological and biological pathways. Translational studies have fast-tracked the application of observations from basic findings to the clinical setting. Amongst the most encouraging methods are transporter and receptor-based imaging, where probes permit the diagnosis of disorders, in addition to the prognostication and evaluation of response to treatments. Precise preoperative staging, surgical preparation, and intraoperative imaging can be achieved using tracers with high sensitivity and specificity. A total of 14 manuscripts were submitted to this special issue, and after rigorous review, 4 were accepted, including one preclinical and three clinical studies.

J. H. Choi et al. created a new approach of radiochemical production via photoactivated reaction to make ^18^F-labeled PET tracers using small molecular and RGD peptides, which can specifically and strongly bind to integrin *α*v*β*3. Integrin *α*v*β*3 is related to angiogenic endothelial and different tumors, including breast, prostate, lung, and ovarian cancers. *In vivo* PET imaging after intravenous administration of an ^18^F-labeled compound to RR1022 sarcoma-bearing Sprague Dawley rats demonstrated a high tumor-to-background ratio, indicating it can be useful for evaluation of tumor angiogenic response.

D. Dai et al. carried out a Phase 2 trial designed to evaluate whether technetium-99m-labeled ethylenedicysteine-glucosamine (^99m^Tc-EC-G) SPECT/CT was noninferior to ^18^F-labeled fludeoxyglucose (^18^F-FDG) PET/CT in 17 patients with confirmed nonsmall cell lung cancer (NSCLC). The authors found 100% concordance between both tracers for primary tumor detection and 70% agreement for metastatic tumor detection, indicating that ^99m^Tc-EC-G SPECT/CT may be a clinically useful radiotracer, warranting the preparation of Phase 3 study.

Y. J. Jeong et al. analyzed dopamine transporters (DATs) with ^18^F-labeled 3-b-(4-iodophenyl)nortropane (FP-CIT) PET on patients with Parkinsonism, cerebellar, and autonomic characteristics in multiple system atrophy with cerebellar ataxia (MSA-C). A total of 49 subjects clinically diagnosed with possible or probable MSA-C were included. The authors found statistically significant differences in postural instability, rigidity, asymmetry, bradykinesia, and specific uptake ratio (SUR) between groups. A subgroup of 22 subjects received dopaminergic medications. Interestingly, all seven subjects with normal exams presented no modification, whereas 10 out of 15 subjects with abnormal exams presented clinical progress.

Finally, D. Dai et al. compared 3D positron emission mammography (PEM) with whole-body PET (WBPET) for patients with breast cancer. A total of 410 women with normal breast, benign tumors, or highly suspicious malignant lesions were randomized at 1 :  1 ratio for imaging with WBPET followed by 3D-PEM or 3D-PEM followed by WBPET. Lumpectomy or mastectomy was carried out on eligible subjects after imaging. The authors reported that 3D-PEM had sensitivity and specificity of 92.8% and 54.5%, respectively, while WBPET had sensitivity and specificity of 95.7% and 56.8%, respectively.

In conclusion, significant developments have been achieved in transporter and receptor-based molecular imaging, and the original articles in this special issue underscore progresses for translation of these promising techniques into the clinic.

*Paulo Henrique Rosado-De-Castro* *David Yang* *Hwan-Jeong Jeong* *Kazuma Ogawa* *Marcos Fabio Henriques DosSantos* *Skye Hsin-Hsien Yeh*

